# Systematic review of Kinect applications in elderly care and stroke rehabilitation

**DOI:** 10.1186/1743-0003-11-108

**Published:** 2014-07-03

**Authors:** David Webster, Ozkan Celik

**Affiliations:** 1 Department of Computer Science, San Francisco State University, San Francisco, CA 94132, USA; 2 Department of Mechanical Engineering, Colorado School of Mines, Golden, CO 80401, USA

## Abstract

In this paper we present a review of the most current avenues of research into Kinect-based elderly care and stroke rehabilitation systems to provide an overview of the state of the art, limitations, and issues of concern as well as suggestions for future work in this direction. The central purpose of this review was to collect all relevant study information into one place in order to support and guide current research as well as inform researchers planning to embark on similar studies or applications. The paper is structured into three main sections, each one presenting a review of the literature for a specific topic. Elderly Care section is comprised of two subsections: Fall detection and Fall risk reduction. Stroke Rehabilitation section contains studies grouped under Evaluation of Kinect’s spatial accuracy, and Kinect-based rehabilitation methods. The third section, Serious and exercise games, contains studies that are indirectly related to the first two sections and present a complete system for elderly care or stroke rehabilitation in a Kinect-based game format. Each of the three main sections conclude with a discussion of limitations of Kinect in its respective applications. The paper concludes with overall remarks regarding use of Kinect in elderly care and stroke rehabilitation applications and suggestions for future work. A concise summary with significant findings and subject demographics (when applicable) of each study included in the review is also provided in table format.

## Introduction

**Figure 1:**
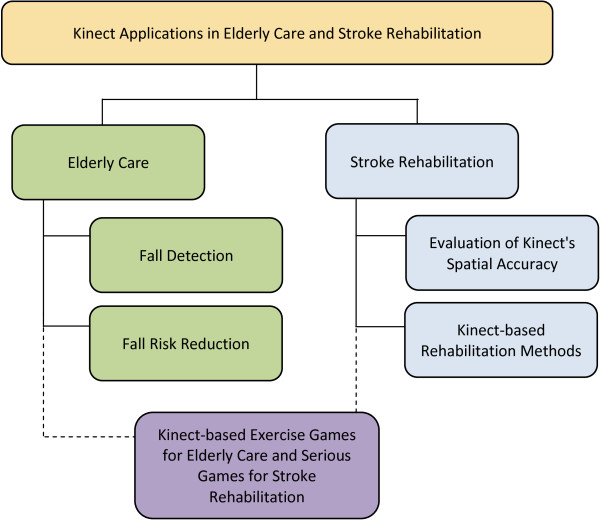
**Manuscript Structure**. Structure of the manuscript summarizing how studies included in this review were grouped together into relevance-based subsections. The Applications in Elderly Care section is comprised of two subsections: 1) Fall detection and 2) Fall risk reduction. The Applications in Stroke Rehabilitation section contains: 1) Evaluation of Kinect’s spatial accuracy and 2) Kinect-based rehabilitation methods. We have included a third section titled ‘Serious and exercise games’ for studies that we believe are indirectly related to the first two sections and present a complete system for elderly care or stroke rehabilitation in a Kinect-based game format. There are many applications of the Kinect in rehabilitative and assistance-based research that, while extremely important, fall outside the scope of this systematic review.

The median age of the general population is projected to significantly rise in the upcoming years [1]. As the elderly population grows in age and size, an increased patient population-based stress will be placed on already overloaded clinics and hospitals. Major contributors to this increase are need of care for the elderly who are healthy to stay healthy (such as physical exercise, fall detection and fall risk reduction) and need for rehabilitation after stroke, for which age is a significant risk factor. The demand for technologically advanced methods of elderly care, which can be accessed at any time and used in a private, home-based setting while still providing rehabilitation instructions and progress tracking, is expected to expand. The Kinect is the forerunner in commercially available hardware upon which development of these methods can be built while simultaneously maintaining affordability for large-scale disbursement [2]. In this paper we present a review of the most current avenues of research into Kinect-based elderly care and stroke rehabilitation systems to provide an overview of the state of the art, limitations, and issues of concern as well as suggestions for future work in this direction. Figure 1 presents the structure of the manuscript, essentially, how studies included in this review are grouped together into relevance-based subsections. **Elderly Care** is comprised of two subsections: 1) Fall detection and 2) Fall risk reduction. **Stroke Rehabilitation** contains: 1) Evaluation of Kinect’s Spatial Accuracy, and 2) Kinect-based Rehabilitation Methods. We have allocated a third section titled ‘Serious and exercise games’ for studies that are indirectly related to the first two sections and present a complete system for elderly care or stroke rehabilitation in a Kinect-based game format. A concise summary with significant findings and subject demographics (if applicable) of each study included in the review is also provided in a table format Tables 1 and 2), to facilitate readers’ access to more detailed information for studies of interest. The remainder of the Introduction section provides a brief overview of each of the three main sections of the paper.

**Table 1:** Overview of studies categorized under the section applications of Kinect in elderly care

**Author**	**Year**	**Population**	**Significant findings**
***Elderly care > Fall detection***			
Kepski et al.	2012	Study type: methodologyParticipants: unspecifiedAge: unspecified	The study utilized a fuzzy inference system which combined data from the Kinect and a wearable accelerometer and gyroscope, and was run on PandaBoard ES in real-time. Unobtrusive fall detection with experimental results indicating high effectiveness of fall detection even in environments lacking visible light were reported.
Planinc et al.	2013	Study type: methodologyParticipants: 2 (unspecified gender)Age: unspecified	Eighteen different sequences consisting of ten true falls and eight non-falls were examined. A comparison to previous fall detection methods, audio-based and 2D sensor-based, using 3D Image Coordinates (IC) and 3D using world coordinates (WC) resulted in: Recall (defined as: $\frac{\text{TruePositive}}{\text{TruePositive}+\text{FalseNegative}}$): IC = 78%, WC = 93%; Precision (defined as: $\frac{\text{TruePositive}}{\text{TruePositive}+\text{FalsePositive}}$): IC = 100%, WC = 100%; F-score (defined as: $2\times \frac{\text{Recall}\times \text{Precision}}{\text{Recall}+\text{Precision}}$: IC = 87%, WC = 96%; True Negative (defined as: $\frac{\text{TrueNegative}}{\text{TrueNegative}+\text{FalsePositive}}$): IC = 100%, WC = 100%, and Accuracy (defined as: $\frac{\text{TruePositive}+\text{TrueNegative}}{\text{TruePositive}+\text{FalseNegative}+\text{FalsePositive}+\text{FalseNegative}}$) IC = 86%, WC = 96%
Rougier et al.	2011	Study type: methodologyParticipants: unspecifiedAge: unspecified	After examining 79 videos: 30 sitting down, 25 falls (including 7 totally occluded), and 24 crouching (including 6 totally occluded), an overall fall detection success rate of 98.7% was observed using the centroid height relative to floor level and velocity of a moving body methodology. All ‘not occluded’ events were correctly classified, but in the case of a total occlusion, utilizing body velocity remains unverified in discriminating a person who falls from a person who brutally sits.
Lee et al.	2012	Study type: researchParticipants: unspecifiedAge: unspecified175 video segments of walking, standing, crouching down, standing up, fallingforward	Algorithm capable of monitoring shadow filled or completely dark environments. The system used three features: bounding box ratios, normalized 2-D velocity variations from the centroids, and Kinect-gathered depth information. The algorithm was then validated by applying it to 175 video segments of walking, standing, crouching down, standing up, falling forward, falling backward, falling to the right, and falling to the left; resulting in an overall accuracy of 97% and a minimal false positive rate of 2%.
Mastorakis et al.	2012	Study type: researchParticipants: 8 (unspecified gender) Age: unspecified	A 3D bounding box methodology was utilized to detect falls using 184 recorded videos: 48 falls (backward, forward and sideways), 32 seating activities, 48 lying activities on the floor (backward, forward and sideways) and 32 “picking up an item from the floor.” Other miscellaneous activities that change the size of the 3D bounding box were also performed (i.e. sweeping with a broom, dusting with a duster). The system was reported as 100% accurate with respect to fall detection with no observed false positives or false negatives; however, due to the unique method of fall detection utilized, if an item (i.e. chair) wasmoved, a new bounding box was created for the item and if it subsequently fell over, a false fall detection could be triggered.
Zhang et al.	2012	Study type: researchParticipants: 5 (unspecified gender)Age: unspecifiedUtilized 200 recorded videos(condition 1 = 100, condition2 = 50, condition 3 = 50.)	System used two models: the appearance model, a method of extracting data from 2D images when subject was out of range of the Kinect’s depth sensing, and the kinematic model using data derived from the Kinect’s 3D world coordinates readings. The model was trained using data captured under three different conditions: 1) less than 4 meters distance - normal illumination; 2) subject in range of depth sensor - without enough illumination; and 3) greater than 4 meters distance - normal illumination. Comparisons were conducted between: falling from a chair (L1); falling from standing (L2); standing (L3); sitting on a chair (L4), and sitting on the floor (L5). Under condition #1, the appearance model resulted in: L1 = 90%, L2 = 60%, L3 = 70%, L4 = 60%, L5 = 100% accuracy, whereas the kinematic model model resulted in: L1 = 100%, L2 = 90%, L3 = 100%, L4 = 100%, L5 = 100% accuracy. Under condition #2, the appearance model resulted in: L1 = 80%, L2 = 30%, L3 = 70%, L4 = 80%, L5 = 10% accuracy, whereas the kinematic model resulted in: L1 = 100%, L2 = 80%, L3 = 100%, L4 = 90%, L5 = 100% accuracy. The appearance approach performed at a speed of 0.0074s. The kinematic approach performed at a speed of 0.0194s.
***Elderly care > Fall risk reduction***			
Parajuli et al.	2012	Study type: methodologyParticipants: unspecifiedAge: unspecified	Four data sets used 1) normal walking; 2) abnormal walking; 3) standing, and 4) sitting. Nine methods utilizing various combinations of the following variables were used: Z-coordinate, absolute height, arms coordinates, and a Support Vector Machine (SVM). Correct detection of normal and abnormal walking, sitting, and standing of a C-SVM (SVM using C-Support Vector Classification) increased from (≈71% to ≈99%) with the use of scaling SVM data. This lead to the conclusion that SVM scaling of data is critical for accuracy within algorithms such as this. Both posture and gait recognition were observed to follow a similar pattern of accuracy.
Gabel et al.	2012	Study type: methodologyParticipants: 23 (m = 19, f = 4)Age: 26 to 56	The study conducted a full body gait analysis of Kinect readings, compared to two pressure sensors (FlexiForce, A2013) and a gyroscope (ITG-3200 by InvenSense4) and resulted in the following (units in ms):
			Left stride: avg strides captured = 1169; mean difference (kinect v. baseline) = 8; SD = 62;
			Right stride: avg collected = 1130; mean difference (kinect v. baseline) = 2; SD = 46;
			Left stance: avg collected = 634; mean difference (kinect v. baseline) = -8; SD = 110;
			Right stance: avg collected = 595; mean difference (kinect v. baseline) = -20; SD = 90;
			Left swing: avg collected = 518; mean difference (kinect v. baseline) = 6; SD = 115;
			Right swing: avg collected = 541; mean difference (kinect v. baseline) = 27; SD = 104;
			Angular velocity of arm resulted in a correlation coefficient between the Kinect-based prediction and the gyroscope-based true value of >0.91 for both arms with an avg difference of (units in °/second): left arm = 1.52; right arm = -0.86 (SD L = 48.36 R = 44.63)
Stone et al.	2011	Study type: methodologyParticipants: 3 (unspecified gender) Age: unspecified18 total walking sequences - two walks were collected for each speed: slow, normal, and fast for each participant.	The calculated percentage difference between the Kinect systems readings and the Vicon system readings for walking speed, average stride time, and average stride length measurements are as follows (Mean (M), Standard Deviation (SD), Maximum (MAX)): Kinect #1 (parallel to sensor): walking speed: M = -4.1%, SD = 1.9%, MAX = 9.6%; stride time: M = 1.9% SD = 2.5%, MAX = 4.1%; stride length: M = -1.9%, SD = 2.5%, MAX = 11.7%. Kinect #2 (away from sensor): walking speed: M = -1.9%, SD = 1.2%, MAX = 4.9%; stride time: M = 0.7%, SD = 1.3, MAX = 8.4%; and stride length: M = -1.1, SD = 2.5, MAX = 9.4%. A secondary artefact noted during this study: typically Kinect-gathered data at a relatively long range becomes unusable; however, utilizing this system, initial data showed little change in accuracy at long range (up to 8.1 meters). A validation of this unusual result has yet to substantiate these initial findings.
Stone et al.	2012	Study type: methodologyParticipants: 7 (m = 4, f = 3)Age: 75–95	Unobtrusively identified walking sequences and automatically generated habitual, in-home gait parameter estimates. The following is representative data for participant 1: Avg. speed (cm/sec): 62.2, computed avg. speed: 61.0; True stride time (sec): 1.17, computed stride time (sec): 1.17; True avg. stride length (cm): 71.6, computed avg. stride length (cm): 70.1; True height(cm): 162.1, computed height (cm): 161.8.
***Elderly care > Kinect gaming***			
Marston et al.	2012	Study type: review	Narrative review of the current technologies viable for game-based solutions to enable enhanced quality of life in the elderly. The use of videogames for health related purposes demands game classification systems which take into account their player-base’s physical, cognitive, and social requirements, which can include a wide range of impairments.
Smith et al.	2012	Study type: review	Provides an overview of the main systems for in-home motion capture and some of the preliminary uses in elderly care, stroke rehabilitation, and assessment and/or training of functional ability of the elderly.
Staiano et al.	2011	Study type: review	Review paper which provides an overview of the measurement capabilities of exergames to derive viable data for clinical data pertaining to physical health, caloric expenditure, duration of use, balance, and other categories of interest.
Tanaka et al.	2012	Study type: review	Comparison of the Kinect, EyeToy, and Wii systems including technical specifications, the motion sensing capabilities of each interface, and the motion required to support therapeutic activity types. Discussion focuses on the unique research implications of using these three motion capture tools.
Wiemeyer et al.	2012	Study type: review	Specific challenges for game design presented: 1) selection of appropriate movements to offer meaningful exercise contexts for older subjects; 2) utilization of devices offering options that combine challenge and support; 3) determining appropriate game-based ‘dosage’; 4) randomized controlled trials to corroborate effects, and 4) development and evaluation of adequate training settings.
Arntzen et al.	2011	Study type: methodologyParticipants: Elderly care workersand one researcherAge: Unspecified	Presented concepts and requirements for developing Kinect-based games for the elderly and presents seven important issues that each game should consider during controller-free game development: visual, hearing, motion, technological acceptance, enjoyment, and emotional response.
Golby et al.	2011	Study type: methodology	The proposed system’s aim is to present occupational therapists with a tool that provides a range of motion analysis which enables gathering of patients’ range of motion from remote locations and the comparison of this gathered data with the range of motion required for a variety of activities of daily living.
Garcia et al.	2012	Study type: methodology	Proposes a system for clinically viable data capture of participants balance level utilizing a Choice Step Reaction Time mini game which requires participants to step on targets in a variety of ways.
Maggiorini et al.	2012	Study type: methodology	Description of a prototype game-based rehabilitation paradigm to enable home-based rehabilitation exercises for the elderly which can be monitored by caretakers of various sorts. The system includes: a distributed software architecture comprising of end systems, elderly users, caretakers, a core server, and a communication system.
Gerling et al.	2012	Study type: researchParticipants: 15 (institutionalizedolder adults, m = 8, f = 7)Age: Range 60 to 90, mean = 73.72 (SD = 9.90)	Investigated how elderly participants responded to game-based gestures. Results were compiled with the positive and negative affect scale (PANAS), mean (M), standard deviation (SD). Overall, the positive emotional affect was slight (before: M = 3.34, SD = 0.64, after: M = 3.88, SD = 0.79, (*t*_11_ = -2.92, p <0.01), whereas the negative emotional affects were less notable: before: M = 1.72, SD = 0.78, after: M = 1.68, SD = 0.86. (*t*_11_ = 0.28, p = 0.79)
Chiang et al.	2012	Study type: researchExperimental Group:Participants: 22Age: 78.55 (± 6.70)	The Vienna Test System, the Soda Pop test, and a Mann-Whitney non-parametric test were used to evaluate beneficial effects of Kinect usage on reaction time and hand-eye coordination. Reaction time (units in milliseconds Vienna Test System): - Experimental group: a median improvement of 167.51, and a decrease in SD of 362.66. - Control group: a median decline of -202.9, and an increase in SD of 183.56.
		Control Group:Participants: 31 Age: 79.97 (± 7.00)	Hand-eye coordination time(units in seconds, Soda Pop test): - Experimental group: a median improvement of 6.01, and a decrease in SD of 0.34. - Control group: a median decline in 1.61, and an increase in SD of 5.49
Chen et al.	2012	Study type: researchExperimental Group:Participants: 21 (m = 3 f = 19) Age: 65–92 Control Group: Participants: 39 (m = 15, f = 24)Age: 65–92	22 out of the 61 participants volunteered to be in the experimental group for a 4-week course of training which involved three 30 minute sessions per week - 5-minute warm up, 20-minute interactive gaming, and 5-minute cool down. Health-Related Quality of Life (HRQOL), SF-8 (Quality Metric) questionnaire of General health (GH); Physical Function (PF); Role Physical (RP); Body Pain (BP); Vitality (VT) Social Functioning (SF); General Mental Health (MH); Role Emotional (RE), was employed in this study and an ANCOVA analysis was done. In the physical component summary of the HRQOL improvements were noted in the categories of general health, physical function, role physical, and body pain (p <0.05). The mental component summary; however, in general showed no significant differences between experimental and control groups (p <0.05). Results are out of 100: Experimental Group: GH = 48.69 to 54.49; PF = 50.73 to 52.34; RP = 51.91 to 52.70; BP = 52.90 to 57.44; VT = 57.16 to 57.04; SF = 52.85 to 55.50; MH = 56.14 to 55.53, RE = 51.19 to 51.83. Control Group: GH = 48.99 to 46.64; PF = 47.76 to 47.90; RP = 48.00 to 47.92; BP = 54.04 to 51.75; VT = 52.51 to 51.12; SF = 47.49 to 47.04; MH = 51.96 to 50.41, RE = 47.10 to 49.67.
Pham et al.	2012	Study type: researchParticipants: 24 (older adults m = 7, f = 17) Age: mean = 74, SD = 6.4	A comparison of button-based, mixed button/gesture-based, and gesture-based controllers was conducted through surveys aiming to identify user preference. The gesture-based controller was most preferred (42%) with the Mixed Controller next (25%) and the button controller last (8%); however, 21% did not care either way, and 4% enjoyed all types equally. Completion times were lower for mixed button and gesture systems, compared to the standalone Button Controller or Gesture Controller (Wilks’ Lambda =.16, F(2,22) = 54.98, p <0.05).
Hassani et al.	2011	Study type: researchParticipants: 12 (m = 5, f = 7) Age: mean = 77.17 (SD = 7.19)range: 71 to 96.	7-point Likert scale (7 - max agreement) on standard deviation for Effort, Ease and Anxiety (EEA) which measures how easily people think they can adapt and learn how to work with the technology, overcoming eventual anxieties and Performance and Attitude (PA) which measures how respondents ‘see themselves’ both practically and socially in the light of the new technology: EEA for Gestures: mean = 6.13, SD = 1.02; EEA for Touch = 6.18, SD = 1.01; PA for Gestures: mean = 6.01, SD 1.43; PA for Touch = 6.00, SD = 1.84.
Sun et al.	2013	Study type: researchParticipants: 23 (m = 12, f = 11) Age: 21 to 30	This study explored how Kinect-based balance training exercises influenced balance control ability and tolerable intensity level of the player. The results showed that varying evaluation methods of player experience could easily result in different findings making it hard to accurately study the design of those exergames for training purposes. This was accomplished by requiring a participant to stand on one leg within a posture frame (PF) and evaluating the resulting balance control ability in both static and dynamic gaming modes using a 6-axis AMTI force plate. The game would move various body-outline shapes toward the player’s avatar, and the player would then have to imitate the body-outline shape in order to pass through it without touching the outline. Force plate data - Fx, Fy, Fz, Mx, My, and Mz - was preprocessed and MATLAB was used for calculations. The following parameters were analyzed: small frame 1-second travel time (SF1S), large frame 1-second travel time (LF1S), small frame 2-second travel time (SF2S), large frame 2-second travel time (LF2S): Mean distance-anterior posterior: SF1S = 0.77(± 0.25); LF1S = 0.70(± 0.18); SF2S = 0.97(± 0.25); LF2S = 0.94(± 0.29) Mean distance-medial lateral: SF1S = 1.98(± 1.16); LF1S = 1.99(± 1.16); SF2S = 1.94(± 1.47); LF2S = 1.72(± 1.32) Total excursions: SF1S = 53.98(± 15.57); LF1S = 53.68(± 17.28); SF2S = 53.68(± 16.32); LF2S = 51.52(± 17.87) Sway area: SF1S = 0.07(± 0.06); LF1S = 0.06(± 0.05); SF2S = 0.06(± 0.05); LF2S = 0.06(± 0.02)

**Table 2:** Overview of studies categorized under the section applications of Kinect in stroke rehabilitation

**Author**	**Year**	**Population**	**Significant findings**
***Stroke rehabilitation > Evaluation of Kinect’s spatial accuracy***			
Pedro et al.	2012	Study type: methodologyParticipants: 1 (robotic arm) Age: N/A	A KUKA robotic arm (precision accuracy of up to 0.05 mm) was utilized for precise movements. The Kinect was attached to this arm and a target was positioned at a static position in the KUKA arm’s work space resulting in Kinect readings with a min error of 0.036 mm, max error of 12.25 mm, mean error of 4.95 mm and standard deviation of 2.09 mm in comparison to the KUKA as a ground truth.
Chang et al.	2012	Study type: methodologyParticipants: 2 (m = 1, f = 1) Age: (unspecified)	Appraised the tracking performance of the kinect specifically for a set of six upper limb motor tasks in regards to a high fidelity OptiTrack optical tracking system consisting of an array of 16 ceiling-mounted cameras. The following motions were utilized: external rotation, shoulder abduction, shoulder adduction (diagonal pull down) scapular retraction, shoulder flexion, and shoulder extension. While a statistical analysis of data captured was not offered, a visual representation demonstrated that data trends for both systems, in regards to hand and elbow represent competitive movement tracking performance, whereas shoulder readings were widely inconsistent. The authors attribute these inconsistent shoulder readings as due to differing methods of motion capture and joint estimation between the OptiTrack and the Kinect. Furthermore, the participants were asked to utilize External Rotation of the shoulder 10 times each, with 5 correct movements and 5 incorrect movements. The Kinect-based game implemented successfully identified all the incorrect movements.
Clark et al.	2012	Study type: methodologyParticipants: 20 (healthy, m = 10,f = 10) Age: 27.1 yr (± 4.5) Height: 173.7cm (± 10.3) Mass: 71.7kg (± 11.0)	Type 2,1 intra-class correlation coefficient difference between Kinect and Vicon Nexus (ICC) and ratio of coefficient of variation difference between systems (CV) was conducted using three postural control tests: a forward reach, a lateral reach, and a one leg standing balance test. The points of examination were of distance reached, trunk flexion angle (sagittal and coronal), and a balance test focused on spatio-temporal changes in the sternum, pelvis, knee and ankle as well as the angle of lateral and anterior trunk flexion. The results demonstrated a very high level of agreement between systems. The following is a sample of reported data (units in mm): Lateral reach: - Sternum: ICC = 0.03, CV = 0.1; Hand: ICC = 0.16, CV = 5.5; Trunk (deg): ICC = 0.01, CV = 0.7; Forward reach: - Sternum: ICC = 0.07, CV = 1.0; Hand: ICC = 0.05, CV = 1.2; Trunk (deg) ICC = 0.00, CV = 0.6; Single leg balance: - For a full-body joint-by-joint char of details see Table one and Table two on page 375 of the study
Obdrzalek et al.	2012	Study type: methodologyParticipants: 5 (unspecified gender) Age: unspecified	Full-body comparison between the Kinect and PhaseSpace Recap for joint position readings of mean difference, standard deviation from mean, and right and left specific measurements. Overall error was typically within sub-centimeter accuracy; however, centimeter level accuracy was also noted on more difficult joint comparisons, such as the hip For detailed results of the comparison based on a front view see Table one on page 5 of the study For detailed results of the comparison based on a 30° view see Table two on page 5 of the study For detailed results of the comparison based on a 60° view see Table three on page 5 of the study
Loconsole et al.	2012	Study type: methodologyParticipants: 1 (healthy, male) Age: 25	This study utilized an L-Exos controller exoskeleton robot arm and a Kinect in order to track a patients upper extremities and objects and examined: 1) light variation: very intensive, medium and low illumination - no substantial differences; 2) occlusions: two objects moved to occlude each other - no adverse effect and both items were correctly recognized again post occlusion; 3) object roto-traslation: rotation and movement of two tracked objects - no substantial error introduced, and 4) accuracy: error was negligible (within 2 cm). Accuracy test starting distances: 500 mm, 700 mm, and 900 mm on the Z axes. The object was moved 10 mm, and then 20 mm, and finally 50 mm along the X and Z axes. The following shows the error introduced by the specified movements on the Z and X axes (all units in mm): 500 distance: +10 mm: Z = 0.1, X = 0.1; +20 mm: Z = 0.3, X = 0.1; +30 mm: Z = 0.5, X = 0.5 700 distance: +10 mm: Z = 0.5, X = 0.2; +20 mm: Z = 0.8, X = 0.2; +30 mm: Z = 1.2, X = 0.5 900 distance: +10 mm: Z = 0.6, X = 0.4; +20 mm: Z = 1.9, X = 0.4; +30 mm: Z = 2.1, X = 0.5
Fern et al.	2012	Study type: methodologyParticipants: 1 (healthy, male) Age: unspecified	Accuracy comparison was done between Kinect (OpenNI and Primesense’s NITE) and a 24 camera Vicon (MX3) system. Movements included: 1) knee flexion and extension; 2) hip flexion and extension on the sagittal plane; 3) hip adduction and abduction on the coronal plane with knee extended; 4) shoulder flexion and extension on the sagittal plane with elbow extended; 5) shoulder adduction and abduction on the coronal plane with elbow extended, and 6) shoulder horizontal adduction and abduction on the transverse plane with elbow extended. Mean Error (ME) and mean error relative to Range of Motion (ROM) was calculated. All error readings for the knee and hip are lower than 10° ranging from 6.78° to 9.92°. Dynamic ranges of motion are between 89° and 115°. ME increases when ROM is higher mainly due to occlusion. Error readings for the shoulder range from 7° to 13°.
***Stroke rehabilitation > Rehabilitation methods***			
Saposnik et al.	2011	Study type: review	A meta-analysis to determine the benefit of VR technology for post stroke upper extremity recovery was conducted and reported improvement of Fugl-Meyer scores and measures of arm speed, range of motion, and force at the ‘Body Structure and Function’ level (of International Classification of Functioning (ICF) [3]). Improvements for the VR-trained experimental groups ranged from 13.7% to 20% vs 3.8% to 12.2% in the non-VR control groups. The ‘Activity’ level of the ICF tests (such as the Wolf Motor Function Test (WMFT), Jebson-Taylor Hand Function Test, and the Box and Block Test) also showed increased results within VR-trained experimental groups from 14% to 35.5% vs 0% to 49% for non-VR control groups. Randomized controlled trials (RCTs) were evaluated using the pooled treatment effect (Mantel-Haenszel (OR)) by using random-effect models to reduce the effects of heterogeneity between studies. The effect of VR-based rehabilitation on motor impairment level once the 5 RCTs were combined was OR = 4.89 (95% CI, 1.31 to 18.3; P <0.02). No significant improvement was noted on the Box and Block Test (2 RCTs; OR, 0.49; 95% CI, 0.09 to 2.65; P = 0.41) or WMFT (3 RCTs; OR, 1.29; 95% CI, 0.28 to 5.90; P = 0.74). When considering observational studies, VR-based intervention affected motor impairment percent improvement by 14.7% (95% CI, 8.7% to 23.6%; P <0.001). VR-based intervention on Jebson-Taylor Hand Function Test, WMFT, and Motor Activity Scale resulted in 20.1% improvement in motor function after VR-based intervention. (95% CI, 11.0% to 33.8%; P <0.001).
Hussain et al.	2012	Study type: methodology	A prototype system SITAR (System for Independent Task-oriented Assessment and Rehabilitation) aimed at delivering controlled, task-oriented stroke therapy in an independent manner with minimal therapist supervision was presented. The SITAR is a tabletop system that has function as an assessment or rehabilitation system for upper extremities. SITAR has three parts 1) a set of intelligent objects for haptic-based patient interaction, 2) a marker-less tracking system using inertial measurement units and the Kinect to track the position of the intelligent objects and the movement kinematics of a subject extremities and trunk, and 3) Kinect-based games to engage and motivate patient participation.
Bo et al.	2011	Study type: methodologyParticipants: unspecified (healthy) Age: unspecified	Study proposed a system which utilized a fusion of Kinect and inertial measurement units (IMU) of gyrometers and accelerometers. Using only IMU sensors, individual errors occur in both gyrometers (accumulated error due to bias) and accelerometers (noise and inertial acceleration peaks). Data was significantly more aligned when a fusion of Kinect and the IMU sensors was used via online calibration; however, the study did not provide quantitative results analysis. A video of the experiment can be found at http://www.lirmm.fr/~hayashibe/IMU/embc2011.wmv
Shiratuddin et al.	2012	Study type: methodology	A framework for utilizing non-contact natural user interfaces for an interactive visuotactile 3D virtual environment system was presented in this study. Utilizing the 3D environment of the Kinect may be an approach which could more accurately stimulate the visual cortex and enable more authentic rehabilitation feedback than the current 2D feedback paradigm, ultimately leading to better outcomes.
Yeh et al.	2012	Study type: methodology	The main objective of the proposed system is to stimulate patient participation in upper limb rehabilitation activities. This is accomplished through various manipulations of a virtual ball that a patient interacts with through control of a Kinect-generated skeleton. In order to target the rehabilitation exercises for clinical purposes, a therapist can control parameters related to the ball (e.g. speed and size).
Da Gama et al.	2012	Study type: methodologyParticipants: 10 (3 physiotherapyprofessionals, 4 healthy adults, and 3 elderly subjects of unspecified sex.) Age: unspecified	The system introduced in this study focused on the guidance and correction of participant movements during motor rehabilitation therapies. The study focused on shoulder abduction using the following requirements: 1) shoulder abduction (angle >= 90°); 2) elbow angle >= 160°; 3) angle between the arm and frontal vector plane of >= 80° and <= 100°; 4) right and left shoulder height (Y coordinate) must be similar (for trunk compensation detection); 5) actual shoulder abduction angle must be higher than it was before; 6) return to starting position. Study examined 50 ‘correct’ movements (e.g. fulfilling all the former requirements) with participant standing, seated, and positioned at different angles in respect to the Kinect sensor. All 50 of these ‘correct’ movements were recognized as correct to the system. 60 unspecified ‘incorrect’ exercises (e.g. not fulfilling all the former requirements) were also performed and recognized as incorrect by the system - including postural compensation. The participants also completed a Likert-scale questionnaire to assess the negative aspects of the system (5 = as strongly agree): size of letters (2.77), information clarity (3.75), and stimulus (3.47). The positive reported aspects were: user satisfaction (4.67), motivation (4.67), the system easiness (4.64).
Pastor et al.	2012	Study type: researchParticipants: 1 (stroke, female) Age: 46	Gameplay involves sliding the impaired limb on top of a transparent support in an attempt to hit various targets. The patients range of motion did not show any statistically significant change before and after system use: Fugl-Meyer score before = 16; after = 16. The patient’s score in game steadily increased during the study; however, the authors note that while the game’s score is proportional to the arm’s movement speed, it does not necessarily correspond to motor recovery.
Frisoli et al.	2012	Study type: researchParticipants: 7 (m = 6, f = 1, three healthy volunteers, 4 chronic stroke patients) Age: healthy = 27 (± 7), stroke = 64.5(±13)	Thisstudy presented a Kinect-based, multimodal architecture for a brain-controlled interface-driven robotic upper-limb exoskeleton with a goal of providing active assistance during reaching tasks for stroke rehabilitation. The individual and aggregated performance of the SVM classifier in both trainings of visual condition only, and robot-assisted sessions were examined. The reported performance was based on the offline evaluation of the SVM classifier on the training set. Averaged Correct Classification Rate (%), Healthy subject (H), Stroke patient (P), All (A): Visual: H = 88.1(±5.9); P = 91.9(±9.3); A = 88.2(±10.4) Robot: H = 81.2(±13.6); P = 90.4(±4.9); A = 89.4(±5.0) All: H = 86.4(±8.3); P = 91.1(±6.9); A = 88.8(±7.9)
***Stroke rehabilitation > Kinect gaming***			
Borghese et al.	2012	Study type: methodologyParticipants: unspecified Age: unspecified	Authors state that the system enables quantitative and qualitative exercise evaluation and automatic game-play level adaptation. Presents two serious minigames: Animal Feeder and Fruit catcher. Animal Feeder offers training for dual tasks management (i.e. using both arms simultaneously for different purposes), and In Fruit Catcher the patient is required to utilize reaching and weight shift without movement of the feet. Also, inappropriate movements issue a warning to the player or, in extreme cases, abort the task when detected as unsafe.
Huang et al.	2011	Study type: methodology	A prototype of a serious game based off Jewel Mine using a Smart Glove that would enable participants to actually reach out and grasp target gems, which are located in a semi-circle above a virtual avatar, and place the gems into a receptacle instead of just touching the gems for collection. This combination would enable concurrent hand and upper limb rehabilitation in one serious game.
Lange et al.	2011	Study type: methodologyParticipants: 23 (m = 19, f = 4)Participants consisted of those with balance issues related to Stroke(n = 10), TBI (n = 4) and SCI (n = 9) and 10 clinicians (m = 4, f = 6)	The study presented and discussed three potential applications of the Kinect. 1) virtual environments, 2) gesture controlled PC games, and 3) a game developed to target specific movements for rehabilitation. A prototype balance-based reaching game was developed based on Jewel Mine; however, only anecdotal qualitative data was presented in that patients had reported that the games were challenging and fun, and they would be likely to use the technology within the clinic and home settings if given the option. Clinicians also expressed excitement about the use of this type of technology within the clinical setting.
Pirovano et al.	2012	Study type: methodology	A low-cost game-oriented platform for patients who would benefit greatly from intensive rehabilitation at home. The system proposed would allow for the patient to continue beneficial physician-controlled rehabilitation exercises through remote monitoring and difficulty adjustments as well as a Bayesian-based adaptation schema for automatic game-based difficulty level adjustments.
Saini et al.	2012	Study type: methodology	The study presented a low-cost game framework for stroke rehabilitation. This program’s goal is to increase patients’ motivation for therapy, and also to study the effects of Kinect-based gaming on hand and leg rehabilitation. Also, game design principles for hand and leg rehabilitation for improving the efficacy of stroke exercise was presented. The proposed framework provides angle based limb representation during exercise to ensure exercises are conducted in a correct biomechanical direction angle lessening the chance of reinjury.
Sadihov et al.	2013	Study type: methodologyParticipants: unspecified amount of therapists and stroke patients with slight impairment. Age: unspecified	Based on the Kinect-based haptic glove algorithms discussed, three rehabilitation game applications were developed: 1) a table wiping game; 2) a meteor deflection game, and 3) a rope pulling game. The table wiping game consists of an avatar-hand used to wipe stains from a table with different vibration patterns being initiated in the worn haptic glove based on the participant’s movements. In the rope pulling game, the participant’s virtual hand is able to grab and pull a colorful rope which can be modified for various feels through different force thresholds and feedback types. The meteor Game allows the player to deflect falling meteors from smashing into a virtual village.
Lange et al.	2011	Study type: methodologyParticipants: 20 (m = 17, f = 4) (stroke, TBI, SCI) Age: unspecified	This study presents a system prototype to assess an interactive game-based rehabilitation tool for balance training of adults with neurological injury and was based off the previously developed Jewel Mine game. A series of interviews with clinicians, researchers and patients suffering from neurological conditions impacting balance was used. Preliminary testing took place in an informal setting and reported results were limited to qualitative data about user perceptions of the technology, motivation to use the technology, and the enjoyment level of the system with no quantitative data presented. The authors note that in general participants found the system usable and enjoyable.
Jiang et al.	2012	Study type: researchParticipants: 3 (upper extremityimpairment, m = 2, f = 1) Age: unspecified	This study presents the following heuristics on selecting gesture patterns for patients with upper extremity impairments based off interviews with subjects with upper extremity impairments and subsequent Borg scale rankings regarding potential movements. The guidelines for gestures selection reported is as follows and were derived using a human-based approach which constructs the gesture lexicon based on studying how potential users interact with each other rather than what would be easy for the system to recognize: (1) Select gestures that do not strain the muscles; (2) Select gestures that do not require much outward elbow joint extension; (3) Select gestures that do not require much outward shoulder joint extension; (4) Select gestures that avoid outer positions; (5) Select dynamic gestures instead of static gestures; (6) Select vertical plane gestures where hands’ extension is avoided; (7) Relaxed neutral position is in the middle between outer positions, and (8) Select gestures that do not require wrist joint extension caused by hand rotation.
Llorens et al.	2012	Study type: researchParticipants: 15 (m = 8, f = 7) Age: 51.87(±15.57)	A Kinect-based stepping exercise game for clinical effectiveness. In this study an exergame was created with an objective of stepping on randomly rising objects that emerged from the floor surrounding the patient. Each participant underwent twenty 45-minute training sessions, which consisted of six 6-minute repetitions with a one minute resting time between repetitions. Each participant completed at least (max 5) sessions per week. Assessment was with the Berg balance scale (BBS) [4]; The balance subscale of the Tinetti performance oriented mobility assessment (POBMAb) [5], and the Brunnel balance assessment (BBA) [6]. Assessment was completed at the beginning with an initial assessment (IA), the end with a final assessment (FA), and 1 month after completion with a final update assessment (FUA). The experimental results demonstrated that virtual training significantly improved time scales in balance recovery for stroke patients. For detailed BBS, POBMAb, and BBA results please see Table two on page 111 of the study.

Users of the Kinect are able to intuitively interact with a computer through various gestures and postures. This natural method of human-computer interaction allows for the development of specialized forms of **elderly care applications** and medical alert support systems. These alert systems focus on reducing the probability a fall incident will occur; a leading cause of injury, emotional distress, and financial burden to the elderly [7-17]. Current prototype alert support systems of fall detection and/or risk reduction show tentative promise of becoming successful tools to extend elderly independent life through accurate fall detection [18-22] and automated gait assessment [23-26].

Ideally, all stroke rehabilitation exercises would be performed with therapist-assisted daily practice; however, the demand this would create for therapists make it logistically impractical and quite expensive. Kinect-based **stroke rehabilitation applications** have the potential to reduce this impracticality through guided interactive rehabilitation and virtualized therapists. The accuracy of the Kinect for clinical use to this end is strong [27-32], supporting the potential for full realization of the latter virtualization paradigm which could make pseudo-therapist assisted home-based rehabilitation a reality [33]. The various Kinect-based rehabilitation methods noted in this review hold great potential not only for supporting accurate completion of rehabilitation [34], but also possibly enhancing clinical record keeping and future medical diagnostic methods [35].

The Kinect also provides a platform for the development of stimulating **game-based applications in both elderly care and stroke rehabilitation**. Serious games offer patients rehabilitation environments which help motivate successful completion of otherwise dreary or demanding rehabilitation regimens [36], whereas the aim of exercise games (also termed “exergames”) is to create stimulating methods of maintaining an active lifestyle tailored to the specific physiological and psychological requirements of the elderly and disabled while providing the benefits of physical exercise routines [37-46].

## Methods

### Inclusion criteria

All peer-reviewed journal and conference proceedings articles published in English, directly (e.g., fall detection) or indirectly (e.g., gait assessment) related to elderly care and stroke rehabilitation and conducted within one of the subfields presented in the previous section, and also in Figure 1, were included in this review.

### Exclusion criteria

As the volume of literature regarding the usage of the Kinect in the fields of interest was not anticipated to be extraordinarily large, but instead not yet aggregated, exclusion criteria for this review was minimal: studies which did not go through a peer-review process for publication or were published in a non-English language, were not directly or indirectly related to elderly care or stroke rehabilitation, or were out of the scope of the subfields mentioned previously, and also in Figure 1, were excluded from the review.

### Information databases & search methodology

The following electronic bibliographic databases were searched: IEEE/IET Electronic Library, PubMed, ACM Digital Library, Computer Science Index, Safari Tech Books Online, and ISI Web of Science. Articles were located using the keyword *kinect* and derived combination sets of the following: *stroke, rehabilitation, gesture, posture, clinical, geriatrics, elderly, ageing, aged, alert, fall, gait, exergame*, and *serious game*. No date range or other limits were imposed during the search. Titles and abstracts of all articles were scanned for relevance and a complete list of possible inclusions was compiled with citation information retained in a LaTeX bibliography file. All relevant papers were then closely examined and if a tagged journal paper was deemed a more complete study of a tagged conference paper, the journal version was included and the conference version was discarded. The literature review was concluded on August 1st, 2013.

### Data collection and presentation

As the topic of this review spans two overarching categories including multiple, not directly inter-related subcategories, data was collected, and is presented, on a by-topic basis through included charts and graphs as well as in-line text.

### Contingency bias

Initially, we planned to assess study quality with the Downs and Black check-list [47]; however, based on the observation that current systems developed utilizing the Kinect are often at an immature ‘proof of concept’ state, this approach was deemed unfruitful. As the Kinect is a newly emerging care and rehabilitation tool, discussing the possibility of various biases within individual papers was determined to be out of the scope of this survey. To this end, all within-scope peer-reviewed studies, including unverified and/or only anecdotally supported, are included in this review.

## Results

**Figure 2:**
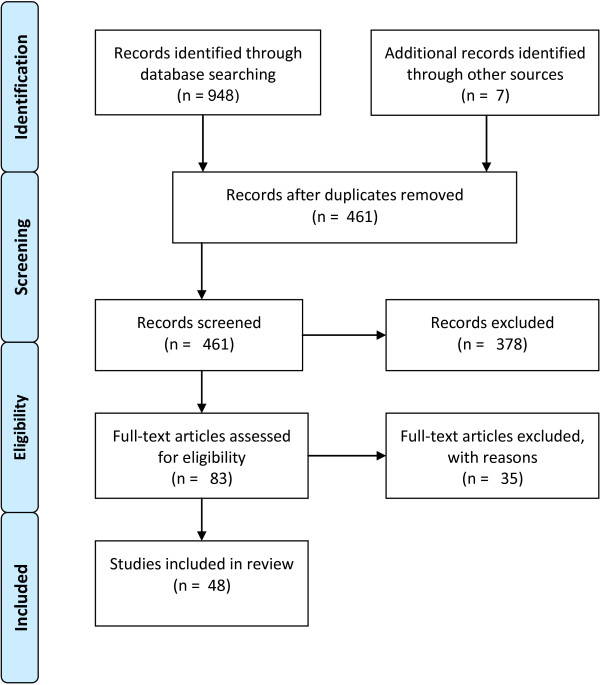
**Study results during PRISMA phases.** Visual representation of the article search and inclusion/exclusion process during different phases of the conducted review process.

Figure 2 summarizes stages of article search and the inclusion/exclusion process. 948 records were located through a search utilizing the methods described in the previous section. An additional 7 records were located by manual searches of relevant research laboratory websites. As the keyword set used was generalized with a large overlap between paper topic areas (e.g. a reference to ‘ageing’ typically would return papers relevant to both elderly care and stroke rehabilitation) a large percentage of initially located papers were duplicates (≈48%). After removal of duplicate papers, 461 records remained. Of these records, 378 were excluded for relevancy issues deduced from titles and abstracts. For example, searching for “Kinect AND stroke” returned publications from a variety of sub-fields unrelated to this review, such as, controller-free exploration of medical image data. 83 full-text articles were then examined and resulted in 35 more exclusions for the following reasons: references to the Kinect was included only in future work, only reference to the Kinect was in citation information, paper was not directly or indirectly related to either elderly care or stroke rehabilitation, paper was not peer reviewed, and/or a more recent and comprehensive version of the study was located in a journal publication. The remaining 50 studies met all criteria of inclusion and an overview of bibliographical information content and main results of all studies are provided in Table 1 and Table 2. To extend the utility of these tables to the reader, each study is categorized as a research, methodology or review paper. In addition to the study type, tables summarize the outcome measures, key findings and human subject demographics (if applicable) of each study.

## Kinect applications in elderly care

In this section we provide a review of applications of Kinect in elderly care grouped under two categories: 1) Fall Detection and 2) Fall Risk Reduction. Technologically advanced alert support systems are a potential avenue of assistance for the independently living elderly person, and these systems could also then be leveraged to produce affordable in-home telerehabilitation methods of care [48]. With falls being a main cause of injury and mortality for the elderly [7-17,49] development of robust, affordable, and widely dispersible in-home **fall detection** and **risk reduction** alert systems is needed and there is significant interest in applications of Kinect to address this need. We refer the reader to Table 1 for a more detailed summary of the studies covered in this section.

### Fall detection

Fall detection has traditionally relied on one or more technologies of panic buttons, audio sensors, physically worn accelerometers or gyroscopes, and/or 2D video capture. Each of these systems comes with inherent limitations: patients with various cognitive deficiencies may be unable to successfully utilize panic buttons, audio sensors are easily overloaded with background noise interference of televisions or music, physically worn devices are cumbersome and wearing them is easily forgotten, and performance of 2D video capture systems significantly degrade in shadow filled or light-less environments [18]. The creation of 3D Red-Green-Blue-Depth (RGBD) cameras is leading to the development of novel alert support system prototypes striving to overcome these previous limitations as well as to enable anonymous privacy preserving fall detection [19,50]. While the added depth field measurement of the Kinect allows for enhancements in previously employed fall detection methods, conclusive evidence of significant improvement in generalized fall detection performance compared to current strategies has yet to be demonstrated. Current studies utilizing the Kinect have been limited to mostly clean environments lacking basic occlusion, and while the results are generally positive, it should be noted that detecting authentic fall occurrences in occluded home environments is a significantly more challenging goal. The Kinect’s initial performance in fall detection remains in need of vindication by more extensive research.

As fall detection systems are typically employed in living environments, variables such as distance from the sensor and illumination are major challenges for current video-based strategies to overcome. Nevertheless, Zhang et al. [19] presented a method of fall detection that continues to perform well in the lack of normal lighting as well as when the participant was beyond the range of depth sensing of the Kinect through RGB video and image processing methodologies. The reported rate of successful fall detection utilizing this method on simulated events performed by five participants was as high as 98%. Similarly, Lee et al. [20] put forth an algorithm capable of monitoring in shadow filled or completely dark environments. This system used three unique features: bounding box ratios, normalized 2-D velocity variations from the centroids, and Kinect-gathered depth information in order to overcome the error introduced by shadows during moving object tracking. An overall accuracy of 97% with a minimal false positive rate of 2% was reported when applying the system to an unspecified ground-truth dataset.

Another current trend of research in RGBD camera-based fall detection support systems is the development of robust systems that can perform unthwarted by occlusion caused by static or mobile scenery. The following three methods aim to overcome this pitfall by utilizing a 3D bounding box, pre-occlusion velocity data analysis for falls which end in an occluded state, and the orientation of a participant’s derived “spine” in respect to a ground plane.

First, Mastorakis and Makris’s [21] bounding box method utilized a participant’s width, height and depth instead of a more standard method of skeletization; calculations derived from a center of mass; or specific measurements of predetermined body points. This approach enabled the system to function fully, without data pertaining scene objects; however, one drawback to this system is that it does not differentiate between bounding boxes created for valid participants and those created for scene objects. If an object falls over, a false positive will occur.

The second method was developed by Rougier et al. [22] and utilized two forms of data: centroid height relative to floor level and the velocity of a moving body. This method allowed for fall detection through use of the former method when there are no significant occlusions, and through utilization of the latter method when the subject is fully occluded after a fall.

The third method, presented by Planinc et al. [18], is a fall detection system that relied on a calculation of a participant’s “spine,” and its orientation with respect to the ground plane. This “spine” is not directly related to the physiological spine, but instead is derived from an analysis of full-body 3D data collected with a Kinect. It can then be assumed that scenarios where this spine rapidly transforms from a state of perpendicular to the ground plane to parallel, and does not return to perpendicular within a specified amount of time, can be considered as a potential fall event.

### Fall risk reduction

Creation of successful algorithms to directly analyze and predict fall potential through real-time Kinect-based systems is a tantalizing, and potentially possible, idea; however, to date, no direct fall prevention algorithms have yet been demonstrated. The existing studies along this line of research are comprised mainly of gait assessment and pre-emptive in-home fall-prevention training exercises, and while gait-based and pre-emptive exercise methods are, at best, only indirectly related to true fall prevention methodologies, they nonetheless have strong potential to contribute to the enabling of accurate fall detection and prevention in the future.

#### Gait assessment to reduce fall events

The studies in this section represent differing approaches in current Kinect-based automated gait assessment methodology research. Stone and Skubic [23,24] conducted a comparison of the Kinect with a ground-truth Vicon system. Gabel et al. [25] and Parajuli et al. [26] each separately demonstrated that a wide range of full-body biomechanical parameters, such as core posture or angular velocities of limbs, were accurately acquirable using The Kinect. Stone and Skubic [23,24] also performed a successful real-world pilot study of their automated gait analysis system in a functional assisted living facility.

Stone and Skubic [24] collected and compared gait related data using the Kinect, a dual web-camera system, and a ground-truth Vicon motion capture system. Not only was the Kinect reported to have sufficient accuracy for clinical gait assessment, it provided significant improvements in foreground capture and overall computational requirements when compared to the low-cost web camera system.

Gabel et al. [25] developed a method of full body gait analysis through the use of Kinect-based data and a multiple additive regression tree algorithm [51,52]. The system monitored the time of the stride, stance, and swing phases of a gait cycle, as well as angular velocities of arm movements; however, measurements of lower limb angular velocities and core posture were also noted to be possible. Prediction of kinematic measurements using these algorithms resulted in a difference of stride measurements between the two systems of 35–71 ms and a correlation coefficient of angular velocity readings between the Kinect and a gyroscope of greater than 0.91 for both arms. Furthering these findings, Parajuli et al. [26] demonstrated that variables of Kinect-based posture and gait recognition can also be accurately acquired (up to 99%) through the use of the specific biomechanic and algorithmic parameters of: height/shoulder width, arms’ coordinates, and c-support vector machine scaling (for an optimal hyper-plane [53]).

Translating Kinect-based gait assessment application from only system accuracy and biomechanic parameter readings to real-world applications, Stone and Skubic [23] developed a system which yielded the simultaneous creation of accurate, autonomous daily in-home gait data profiles of multiple residents of an assisted living facility. These profiles were then examined and reported as containing sufficient parameters for diagnostic use.

### Limitations of Kinect-based elderly care

As the Kinect is a relatively new piece of hardware, establishing the limitations of the sensor within specific application scenarios is an ongoing process. Nevertheless, we provide a list of current limitations of Kinect that we noted based on our review of applications in elderly care systems. 

1. Current Kinect-based fall risk reduction strategies are derived from gait-based, early intervention methodologies and thus are only indirectly related to true fall prevention which would require some form of feedback prior to a detected potential fall event.

2. Occlusion in fall detection algorithms, while partially accounted for through the methodologies of the various systems discussed, is still a major challenge inherent in Kinect-based fall detection systems. Current strategies focus on a subject who stands, sits, and falls in an ideal location of the Kinect’s field of vision, while authentic falls in realistic home environment conditions are more varied, therefore the current results should not be taken as normative.

3. The Kinect sensor must be fixed to a specific location and has a range of capture of roughly ten meters. This limitation dictates that fall events must occur directly in front of the sensor’s physical location. While it has been noted that a strategically placed array of Kinect sensors could mitigate this limitation [32,54], a system utilizing this methodology has not yet been implemented and evaluated.

4. Without careful consideration of the opinions of a system’s proposed user base, concerns regarding ubiquitous always-on video capture systems, such as the Kinect, may inhibit wide-scale system adoption. During the review, it was noted that research related to the reception of alert support systems is at an early phase, likely due to in-home hardware previously being cumbersome and expensive. With the Kinect having the potential to be widely disbursed in in-home setting monitoring systems, this avenue of research has become more viable and relevant [55-57].

## Kinect applications in stroke rehabilitation

In this section we provide a review of applications of Kinect in stroke rehabilitation grouped under 2 categories: 1) Evaluation of Kinect’s Spatial Accuracy, and 2) Kinect-based Rehabilitation Methods. These categories follow the trend of the literature to first evaluate the Kinect sensor as a clinically viable tool for rehabilitation. Motor function rehabilitation for stroke patients typically aims to strengthen and retrain muscles to rejuvenate debilitated functions, but inadequate completion of rehabilitation exercises drastically reduces the potential outcome of overall motor recovery. These exercises are often unpleasant and/or painful leading to patients’ tolerance for exercise to decrease as indicated by Dobkin et al. [58]. Lange et al. [59] noted that decreased tolerance or motivation often lead to intentional and unintentional ‘cheating’ or, in the worst case scenario, avoidance of rehabilitation exercises altogether. The Kinect may contain the potential to overcome these barriers to in-home stroke rehabilitation as an engaging and accurate markerless motion capture tool and controller interface; however, a functional foundation of Kinect-based rehabilitation potential needs to be established focusing on the underlying strategies of rehabilitation schemas rather than the placating effects offered by serious games. We refer the reader to Table 2 for a more detailed and comprehensive summary of articles focusing on Kinect-based stroke rehabilitation.

### Evaluation of Kinect’s spatial accuracy

Advances in the field of gesture controlled user interfaces have only recently erupted in popularity and functionality due to the development of new, affordable computer vision technology. Historically, a majority of research in gesture controlled virtual reality interfaces has been focused on upper limb rehabilitation [60], usually utilizing hand gestures that required various bulky and impractical designs [61,62]. With ubiquitous computing hardware advances, such as the Kinect, current research is rapidly migrating toward a more compact and direct human communication method of gestures and gesture patterns. Through these advances, the advantages virtual reality systems have previously shown to offer in a clinical setting and novel home-based stroke rehabilitation paradigms, are becoming feasible. However, before Kinect-based motion capture systems can be deemed useful, the spatial accuracy and resolution of the heart of these systems-Kinect-gathered data-needs to be thoroughly examined.

The following studies focus on evaluating the accuracy of the Kinect, and when placed under scrutiny, the Kinect has been found, in general, to carry significant potential for a cost-effective motion capture system for rehabilitation. Chang et al. [27] and Loconsole et al. [30] specifically examined accuracy of Kinect in recording upper extremity movements, whereas a whole-body postural evaluation approach was taken by Clark et al. [28], Fern et al. [31], and Obdrzálek et al. [29]. Furthermore, in an attempt to remove the variability introduced through human subjects during accuracy evaluations, Pedro et al. [32] utilized a robotic arm. As the volume of accuracy evaluation studies focusing on specific postures or scenarios is large, we refer the reader to Table 2 for more detailed reports and accounts of these individual studies. In the remainder of this section, we provide a summary of most prevalent results of Kinect accuracy evaluation studies.

Research related to the Kinect’s ability to accurately capture upper extremity movements is consistently reported as sufficient for clinical use with regards to the elbow and wrist joint tracking; however, mixed results have been reported for the shoulder. Loconsole et al. [30] leveraged a setup containing real, rather than solely virtual, objects, and while some accuracy variation in all joint trajectories was noted depending on the object’s distances on the Z axis (towards the object from the camera) and X axis (horizontal, sideways from the camera), all tests - including those for the shoulder joint trajectories - resulted in readings of within 2 cm of the correct/baseline values. This was reported as well within the limits of rehabilitation needs. Chang et al. [27]; however, observed acceptable wrist and elbow joint tracking, but the shoulder trajectory readings were found to be widely inconsistent. The authors attribute these inconsistencies to differing methods of motion capture and joint estimation between the ground-truth OptiTrack system and the Kinect. On the other hand, even with these inconsistencies, when participants were asked to utilize external rotation of the shoulder during game play, the system successfully identified all non-external rotation movements performed as incorrect.

Clark et al. [28], Obdrzálek et al. [29], and Fern et al. [31] concluded that, in general, the Kinect has sufficient accuracy for the assessment of whole-body kinematics for postural control and diagnostic purposes. The notable issues of concern with regards to Kinect-based accuracy values between these three studies ultimately related to one of two things: self-occlusion errors (which can be caused by the angle between a participant and the sensor, specific movements such as placing a hand on one’s lumbar spine, or when the scene contained non-participant objects such as wheelchairs or walkers) or proportional biases which, when observed, always occurred in complex embedded systems such as the pelvis, sternum or shoulders.

In order to simplify the method of verifying the Kinect’s accuracy for rehabilitation and to avoid the influence of various errors introduced by human biomechanics, Pedro et al. [32] utilized a ‘points of interest’ approach rather than a whole-body kinematic analysis. Under this methodology, the Kinect readings had good repeatability in both X and Y axes, whereas repeatability worsened as the distance to the ‘point of interest’ (Z axis) increased. Data gathered in this study showed that the average of the standard deviation of spatial error increased quadratically with distance; however, even with this limitation it was noted that the Kinect retained a sufficient level of accuracy at manufacturer recommended distances for use in rehabilitation applications.

In an attempt to further improve accuracy of Kinect, including lessening occlusion error or enhancing fine motor control capture, the use of the Kinect together with various inertial sensors has sparked interest. Hussain et al. [63] made use of Kinect-monitored manipulation of specially designed intelligent objects (i.e a can, a jar, and a key-like object embedded with inertial sensors) for fine motor control diagnostics of the hand and wrist. This allows for a virtual environment to monitor the location and kinematics of both the user and the objects manipulated by the user. A variety of hand-held objects very similar if not identical to those prototyped by Hussain et al. are utilized in current, widely used stroke impairment classification tests [64]. Data fusion systems of this type have the potential to enable low-cost home-based stroke impairment quantification tests for both gross and fine motor skills.

As noted by Obdrzálek et al. [29], the Kinect does not perform well at skeletizing positions under significant participant occlusion, or non-participant object interference. When compensation for this deficiency is required for more specific applications, skeletization based on a fusion of Kinect-gathered and worn inertial sensor data show promise for accurate data collection. Bo et al. [65] used inertial motion sensing units composed of 2-axis gyrometers, 3-axis accelerometers, and the Kinect (using Primesensor NITE Middleware) to support accurate Kinect-based data capture with significant occlusion-based error reduction. This error reduction was accomplished by utilizing Kinect readings, when available, as a method of inertial sensor calibration, and inertial sensor estimations when Kinect readings are unavailable due to occlusion.

### Kinect-based rehabilitation methods

The ultimate goals of validating Kinect’s accuracy for rehabilitation are diagnostics (quantifying motor function improvement level of patients) and development of home-based rehabilitation protocols. In this section, we provide a summary of studies that focused on Kinect’s applications to pursue these goals, and their results on provisionary physical and mental benefits.

Virtual reality-based rehabilitation offers a a highly interactive system with many documented benefits specifically to stroke patients [66], and a large variety of Kinect-based approaches of stroke rehabilitation have recently come to the forefront. From Da Gama et al. [67] Pastor et al. [68], and Yeh et al.’s [69] virtual exercise guide and game-based rehabilitation systems, to Shiratuddin et al.’s [70] interactive visuotactile 3D virtual environment, and Frisoli et al.’s [71] multi-modal architecture for brain-controlled interface-driven robotic upper limb exoskeleton, there is a broad range of potential Kinect-based applications.

Promoting proper form/posture, repetition, and enjoyability of stroke-based impairment rehabilitation exercises support and foster motor recovery. Toward enabling proper form, Da Gama et al. [67] developed a system which focuses on the guidance and correction of targeted upper extremity exercise movements. This system monitors and corrects inappropriate postural compensation, a common but discouraged strategy during stroke rehabilitation. Yeh et al. [69] proposed a system that, through the manipulation of varied virtual balls, aims to entertain a patient who has to perform repetitive and what would otherwise be dull exercises. The enjoyability of a task is commonly linked in part to personal performance, and building on this premise, Pastor et al. [68] presented a game-based system where the level of difficulty can be personalized to the patient’s specific impairment-related needs through explicit/direct parameter adjustments or based on performance during game play.

Hints of various multidisciplinary directions Kinect-based research is expanding toward can be seen in the more intricate applications of the Kinect presented by Frisoli et al. [71] and Shiratuddin et al. [70]. Frisoli et al. proposed a Kinect-based, multi-modal architecture for a brain-controlled interface-driven robotic upper limb exoskeleton with a goal of providing active assistance during reaching tasks for stroke rehabilitation. At the level of action planning, the patient’s intention to move towards an object is acquired through a Kinect-based vision system that identifies and tracks physical objects, and an eye-tracking system. At the level of action, brain activity is analyzed during motor imagery and controls the exoskeleton accordingly. Experimental results demonstrated that operating the exoskeleton movement through brain-computer interaction was successful with a classification error rate of 89.4 ± 5.0%. Shiratuddin et al. also proposed a unique framework which utilizes non-contact natural user interfaces, such as the Kinect, in an interactive visuotactile 3D virtual environment rehabilitation system.

These initial benefits, system ideas, and hints toward future research demonstrate a strong potential for fruitful Kinect development, as well as enhancing previously developed widely used out-patient rehabilitation services [72]. These initial studies, by and large, present positive results; however, the potential impact Kinect-based rehabilitation may have on future paradigms is only currently emerging and it is difficult to predict how widespread such systems will become. Their use depends largely on their success in practical implementation, validation of benefits and acceptance by users.

### Limitations of Kinect-based stroke rehabilitation

The current stage of Kinect-based rehabilitation literature is lacking in reported functional and validation data because a majority of systems are only at a proof of concept stage. The following over-arching limitations have been derived from the current state of the literature: 

1. While initial Kinect-based comparisons with research grade motion capture systems demonstrated highly correlated trends and reasonable accuracy, a majority of evaluation studies focused only on sets of gross movements that are advantageous for Kinect. Evaluation of more realistic and/or specific diagnostic movement sets are still needed.

2. The Kinect is unable to accurately assess internal joint rotations of the shoulder and instead utilizes a much less clinically viable single-point estimation. Use of the Kinect for specific shoulder-based functionality requirements have yet to be shown to be clinically viable.

3. Rehabilitation goals which include fine motor skills can not be captured by the Kinect alone; however initial studies suggest fusion systems of Kinect and inertial sensors can be a feasible alternative.

4. Kinect systems are usually not suitable for severely disabled patients, as gross movements that remain extremely small in their entirety are difficult for the Kinect to accurately capture.

## Serious and exercise games

Historically, virtual reality rehabilitation has always been a promising field with an infeasibly high price tag for mass implementation [73]. Tanaka et al. [2] note that recent research, focused on hardware and software, has lead to Sony’s Eyetoy, Microsoft’s Kinect, and Nintendo’s Wii becoming the top three market contenders as tools for low-cost virtual reality rehabilitation platforms. This initial comparison study concluded that the Kinect is the forerunner of these top three tools, citing three main reasons: 1) the Kinect provides the most natural form of human-computer interaction; 2) the Kinect is the most feasible technology for a widely dispersed system of elderly exergaming as it utilizes vision-based data capture and requires no extraneous hardware, and 3) the freedom of controller-free data acquisition and ease of developer access to the Kinect platform required for the development of novel and high quality rehabilitation systems and exergames.

The benefits of focused physical tasks and exercise to stroke victims and the elderly have a rich and well documented history [37-46]. Growing out of this solid foundation, Kinect-based gaming has notable potential to create a low-cost and enjoyable exercise setting while simultaneously gathering quantitative data related to rehabilitation progress, general caloric expenditure, and aerobic activity [74,75]. Current Kinect-based gaming research consists of exergames and serious games. Exergames (a term for exercise games) aim to combine natural human movements and the entertainment of video games to promote elderly exercise and enable built-in unobtrusive diagnostics, whereas serious games intend to simultaneously rehabilitate motor-impaired users while evaluating patient progress and monitoring for potential patient injury. In this section, we provide a review of studies and their results involving use of Kinect in serious and exercise game applications. Again, we refer the reader to Tables 1 and 2 for more comprehensive summaries of articles focusing on this topic.

### Design considerations

In the past, game development has focused on utilizing a player’s cognitive abilities to create an enjoyable experience. The physical dimension of serious and exergames has added an extra challenge to game development while simultaneously enabling video games to find alternative applications in facilitating general and rehabilitation-based exercises. The majority of current design considerations focus on accessibility challenges caused by software development decisions [76] and hand-held and floor-based physical devices such as the Wii, EyeToy, and Dance-Dance Revolution modifications, in addition to Kinect [77,78]; however, our discussion here focuses mainly on design considerations for Kinect-based systems.

In their comparison of elderly preferences between button-based, mixed button/gesture-based, and gesture-based controllers for game play, Pham et al. [79] observed that Kinect-based controller-free design carries the benefit of being the preferred choice of the elderly. Three main findings were reported: 1) older participants preferred less or no physical controlling devices (42% prefered gesture-only, 25% preferred mixed, 8% preferred button-only, and 25% had no preference); 2) the requirement of larger physical movement of the Kinect did not stop it from being the most attractive system, and 3) older adults perceived the need to develop their knowledge and skill further for complete use of the Kinect.

Arntzen et al. [80] examined the physical and cognitive requirements of game design targeted for elderly players based off of interviews and a literature review. The resulting design considerations for controller-free game development were compiled to define seven categories: 1) visual; 2) hearing; 3) motion; 4) technological; 5) acceptance; 6) enjoyment, and 7) emotional response. Furthermore, they suggested an iterative approach to game development, in which a preliminary assessment should be done with patients using traditional games, and results of the assessment should inform refinement and definition of requirements. Once a game prototype is developed, another assessment should be conducted on the usability of the system by those with age related cognitive and/or physical disabilities. Gerling et al. [81] also proposed an iterative method of game development and noted that while age-related visual, hearing, and motion impairments can be accounted for during design, it is advisable to conduct multiple stages of user-feedback driven design prototypes in order to accommodate more specific impairments as well as to ensure user approval in the cognitive categories of technological acceptance, enjoyment, and emotional response.

McNiell et al. [82] offered suggestions for future rehabilitation game development based on previous work and a literature survey. They highlighted that the response to failure and poor performance, in any game, is integral to its use by a player base, and hence should be taken as an important consideration. They suggested that including appropriate positive and encouraging feedback mechanisms are necessary tools to overcome the discouragement that system unfamiliarity and poor motor skills will inevitably cause during use of a serious or exercise game.

Jiang et al. [83] suggested a number of heuristics for selecting Kinect-based gesture patterns specific to patients with upper extremity impairments. The guidelines for appropriate gesture selection were derived using a human-based approach which constructs the gesture lexicon based on studying how potential users interact with each other rather than what would be easy for the system to recognize. These guidlines included the following: (1) Select gestures that do not strain the muscles; (2) Select gestures that do not require much outward elbow joint extension; (3) Select gestures that do not require much outward shoulder joint extension; (4) Select gestures that avoid outer positions; (5) Select dynamic gestures instead of static gestures; (6) Select vertical plane gestures where hands’ extension should be avoided; (7) Relaxed neutral position is in the middle between outer positions, and (8) Select gestures that do not require wrist joint extension caused by hand rotation.

### Exercise games

The Kinect is not unique in its ability to provide vision-based data capture capable of supporting gaming paradigms; however, current research grade multiple camera motion capture systems are typically expensive, difficult to set up, and require a knowledgeable operator. The Kinect does not suffer from these challenges, and with its low-cost, leading the emergence of natural gaming paradigms and development of targeted exercise games (the term “exergames” is also commonly used) in a variety of areas.

Pham and Theng [79] demonstrated an interesting interaction between participant performance and preferences. When given the choice among a button-based system (Wii), a system that fused button-based and vision-based (Wii/Kinect), and a solely vision-based (Kinect) system, the majority of elderly participants gravitated toward the Kinect-only system; however, performance measurements suggested that a fusion system of physical buttons and Kinect resulted in higher performance. Two main benefits cited for this general preference of the Kinect-only system were the remote range provided and the more comfortable method of human-computer interaction. Hassani et al. [84] noted that this more comfortable interaction method was especially observed in frail or partially disabled participants who did not desire to get up and walk toward a computer screen. Furthermore, a completely home-based system, as described by Maggiorini et al. [54], would be ideal for a game-based exercise and rehabilitation paradigm.

Gerling et al. [85] conducted two studies to examine the use of Kinect as a human-computer interface for older adults. In the first study, an evaluation of elderly participants’ performance using a set of gestures developed with the aid of a physical therapist was performed. Based on the resulting limitations observed in movement patterns, an exergame was designed targeting institutionalized elderly participants. The second study then investigated how participants responded to the derived game-based gestures, and concluded that Kinect-based gaming has a positive effect on users emotional well being. Sun et al. [86] developed a Kinect-based exergame which allowed players to participate in interactive balance exercises with visual feedback, and explored how Kinect-based balance training exercises influence the balance control ability and the tolerable intensity level of a player. The game would move various body-outline shapes toward the player’s avatar, and the player would then have to imitate the body-outline shape in order to pass through it without touching the outline. As differing player experience evaluation methods resulted in different findings, it was concluded that care must be taken while deciding on which evaluation methods are to be employed within game design. Chiang et al. [87] examined the health benefits of somatosensory video games specifically related to reaction time and hand-eye coordination on institutionalized older adults confined to wheelchairs. “Follow the Arrow”, “Matchmaker”, and “Mouse Mayhem” –three previously developed games– were modified for the Kinect and then utilized to gather participant related data. A significant decrease in the mean and standard deviation times from start to target were noted in the experimental group (which received Kinect-based training) while the control group lacked any observable improvement. Chen et al. [88] presented a study which attempted to quantify the health benefits of Kinect-based somatosensory video games to older adults with disabilities. Various physical benefits were noted throughout the study; however, mental benefits, in general, showed no significant differences between experimental and control groups.

Each of these systems concluded with overall positive results and demonstrated that Kinect-based gaming can significantly improve quality of life using a variety of measures, such as participant’s emotional state [85], physical function, level of body pain [88], visual performance skills, reaction time, and hand-eye coordination [87]. A caveat to these positive results can be seen in that evaluation methods based specifically on player experience can result in notably different outcomes. Thus exergames for training purposes strongly building on player experience as a metric should be designed with care [86]. Also, to understand the efficacy of somatosensory video game intervention, more rigorous examination needs to be conducted in order to strengthen these initial results.

### Serious games

The physical changes that accompany ageing affect a wide range of functions, including sensory-perceptual processes, motor abilities, response speed and cognitive processes [89]. Research on the efficacy of serious games to retain and rehabilitate optimal abilities have been limited mainly to qualitative studies with small sample sizes and focusing on a variety of controllers and inertial sensor systems [33]. This limitation can also be seen in the literature for current Kinect-based serious games, as the majority of studies have not yet moved beyond initial game design and development.

For many stroke patients, balance and weight shift management constitute a risk of secondary injury [90]. Lange et al. [91] prototyped a serious game based on their Flexible Action and Articulated Skeleton Toolkit (FAAST) which enabled a Kinect-based system to run Jewel Mine; a balance rehabilitation game which encourages the user to reach outside of their base of support. Based on discussion and technical support from Lange et al., Huang et al. [92] proposed a smart glove extension to their system for concurrent hand and upper limb rehabilitation by requiring a player to actually grasp the virtual gems and place them into a receptacle instead of just hitting them. Also utilizing the fusion of a Kinect sensor and a haptic glove, Sadihov et al. [93] developed three minigame applications to offer a variety of game play, motor requirement, and difficulty options: 1) a table wiping game; 2) a meteor deflection game, and 3) a rope pulling game. This methodology of developing multiple minigames for maximum variety of play and motor tasks was also employed by Borghese et al. [34] in the development of Animal Feeder, which offered training for dual task management, and of Fruit Catcher, a game which required reaching and weight shift techniques to be employed without movement of the feet. Borghese et al.’s system also carries the potential to utilize Kinect-obtained information about a patient to both fine tune rehabilitation game parameters and to assess patients’ improvement.

Crosbie et al. [60] noted that friendly competition built into a stroke-based serious game can increase social activity and enjoyment; however, it also is feasible to anticipate a patient inadvisedly attempting to ‘win’ or surpass a set ‘high score’ becoming physically exhausted from over use - especially in systems based on remote monitoring and lacking direct clinical monitoring [35]. This competitive aspect of human nature has the potential to drastically hamper successful rehabilitation. As a solution to this problem, Saini et al. [94] proposed the “watch dog” monitor in order to prevent overuse or overexertion injuries. This monitoring system alerts a user if a game maneuver they execute goes beyond therapist-recommended kinematic limb and body angle limits and ensures that users will not exercise for periods of time so extended as to be counter-productive to rehabilitation goals.

Concerning lower-limb rehabilitation, Llorens et al. [95] developed a serious game which functioned by estimating participants’ foot locations and then creating two virtual feet on a screen with the game objective of using these virtual feet to step on randomly rising targets that emerged from the floor. The results of this follow-up study involving chronic stroke patients showed improvement on the Berg Balance Scale [4] of 49.00 to 52.13 which was noted by the authors as surpassing standards of post-stroke improvement in functionality previously established by Liston et al. [96].

Throughout all of these developed systems, one thread of consistency is the positive reception by players and initial rehabilitation results. The overall view in the literature related to serious games is positive; however, the level of current confidence is almost unanimously recognized as tentative and needing further study.

### Limitations of current Kinect-based serious and exercise games

The limitations specific to Kinect-based serious and exercise gaming applications, considering the requirement of clinical data capture for specific limb movements; specific player-base-desired design considerations; varying levels of limb impairment, and previously defined serious and exergame-based requirements, can be summarized as the following: 

1. Any games designed specifically for diagnostic usage are limited to non-occluding movements. This implies that standard stroke impairment level tests requiring extensive occluding movement sets may be untenable for a Kinect-based system to capture.

2. Diagnostic potential for extremities is limited to gross movements, as fine movements of the hand and foot are currently outside the Kinect’s capture sensitivity.

3. Games targeted at rehabilitation may be prone to “cheating” (e.g. excessive, unnatural and counter-productive trunk-based compensation).

4. Appropriate response to failure and poor performance, if not accounted for during game design, can inherently limit positive outcomes due to demotivation/discouragement resulting in less frequent/consistent use of the system.

5. The current benefits of Kinect-based gaming have only tentatively been studied with mainly short term and small sample sized studies. Data to date should be seen as initial results, and not normative.

## Discussion

As Kinect-based elderly care and stroke rehabilitation research is in its infancy, a majority of the data acquired is qualitative with a focus on self-report and personal opinion. Compounded with this observation is the fact that the data is derived only from small groups of participants, it is anything but normative and should be viewed as tentative initial results. Filling the deficiencies of quantitative and large population based research thus remains a potentially fruitful and necessary avenue of future work. The current applications of Kinect in elderly care seem to be at a more mature state than those of stroke rehabilitation; however, even with the current deficiency, we believe that the Kinect carries the potential to become a future cornerstone of widely dispersed care and rehabilitation systems.

With regards to fall detection and fall risk reduction applications, each of the current technologies of panic buttons, audio sensors, body-fixed systems, 2D and 3D video capture systems comes with inherent physical and financial limitations. While the Kinect does not render any of these technologies obsolete, and comes with its own limitations, its unique aspects may enhance current systems, or potentially be the foundation for newly developed functionalities. 

1. The autonomous nature of the Kinect allows for fall detection without requiring a user to physically trigger a panic button system, or the wearing of cumbersome physical devices, which can often be forgotten.

2. The Kinect comes with built in directional sound capture capabilities, possibly enabling it to be used as, or in unison with, current audio sensor-based systems. This is a functionality of the Kinect not yet studied.

3. The added third dimension depth field measurement offered by the Kinect requires less overhead than current methodologies, and enables the development of more accurate methods of fall-related image processing to be developed.

4. The low-cost, marker-less, and widely dispersible nature of an already commercially available gaming system immediately enables the current clinical virtual reality-based rehabilitation methodologies to be rapidly relocated to individual home settings.

While questions such as participants with what level of impairment most strongly benefit from using the Kinect, how to build an ideal exercise routine for preventative or rehabilitation needs, which is the most performant fall detection algorithm, or how to most efficiently leverage all the various capabilities of the Kinect are still unanswered, the overarching conclusions of this review point toward the Kinect as a promising technology for a wide range of elderly care and stroke rehabilitation applications and need for studies involving larger participant pools to establish reliability and validity.

### Remarks and suggestions for future work

We have compiled the following list based on our review of the current state of Kinect-related elderly care and stroke rehabilitation literature. It contains our remarks as well as suggestions for relevant future research. 

1. A majority of applications in elderly care and stroke rehabilitation require a robust and easily manipulated user interface which at present cannot be readily found in current commercial systems. When developing exercise games, serious games, and applications of Kinect-based rehabilitation, it is vitally important to remember that repurposing a technology initially intended for a younger and healthier audience requires careful adherence to new design strategies focused on both the physiological and psychological requirements of aging and injured users. Therefore we suggest that multidisciplinary research teams involving engineers as well as clinicians, human factors experts and cognitive psychologists would be best positioned to tackle the challenges that such game development efforts would entail.

2. A critical validation step for Kinect-based applications to both fields is a focused experimental evaluation of the accuracy and latency of the motion capture data obtained from Kinect in comparison with a research grade motion capture device and statistical evaluation of this data for specific diagnostic potential. The effects of distance from the Kinect sensor on gathered data is another important consideration. In order to verify that the Kinect has the potential to make therapy financially accessible and medically beneficial to a large population of elderly and stroke patients, more targeted studies involving relevant rehabilitation and preventative exercise movements, are needed.

3. Kinect applications may have the potential to simultaneously achieve care or stroke related goals while capturing real-time, clinically viable data for injury risk evaluation. The real-time data gathering aspect of the Kinect has yet to be satisfactorily examined as a majority of documented work focuses solely only providing assistance or motivation to the user while ignoring this important potential function.

4. As alert systems potentially gather data in an always-on fashion even what might normally be considered mundane activities can then turn into potential privacy infringements. Because of this potential problem, the reception and concerns of the elderly related to always-on systems require a thorough and careful examination.

5. Game content specifically designed for aging and/or injured users must simultaneously allow for high standards of captivating game play and long-term enjoyment potential while maintaining seamless methodologies of adaptation and monitoring of players needs, which are critical characteristics of a successful low-cost home-based rehabilitation paradigm.

6. Generic game play may be unsuitable for many patients with secondary disabilities not solely defined by motor function. Strategic adaptation schemas for game play are necessary traits as complex demands for speech, memory, or cognition patterns significantly reduces the potential for games with large and diverse player bases.

7. The stimulating aspect of exergames and serious games, while being beneficial in patient motivation, should be closely monitored as to provide an exercise or rehabilitation environment that discourages overexertion injuries in both individual movements and length of play.

8. Assumptions of rehabilitation success should not be based on in-game score improvement as arbitrary scores do not necessarily correlate with actual functional improvement, and evaluation methods based specifically on player experience can result in notably different outcomes, therefore using player experiences or in-game scores as metrics should be avoided.

## Conclusions

In this paper, various applications of the Kinect in the fields of elderly care and stroke rehabilitation have been examined. We have classified these applications into the groups of (1) Fall Detection, (2) Fall Risk Reduction, (3) Evaluation of Kinect’s Spatial Accuracy, (4) Kinect-based Rehabilitation Methods, and (5) Serious and Exercise Games - serious games for stroke rehabilitation and exercise games for the elderly. While only in its initial stages of development, the Kinect already shows notable potential in making therapy and alert systems financially accessible and medically beneficial to a large population of elderly and stroke patients; however, some significant technological limitations still present are: a fixed location sensor with a range of capture of only roughly ten meters; a difficulty in fine movement capture; shoulder joint biomechanical accuracy, and fall risk reduction methodologies that only utilize indirect, gait-based pre-emptive training. The directions for future work are vast and have promise to enhance elderly care; stroke patient motivation to accurately complete rehabilitation exercises; rehabilitation record keeping, and future medical diagnostic and rehabilitation methods. Based on our review of the literature, we have reported a summary of critical issues and suggestions for future work in this domain.

## Abbreviations

CVA: Cerebrovascular accident; RGBD: Red-green-blue-depth; FAAST: Flexible action and articulated skeleton toolkit.

## Competing interests

No financial or non-financial competing interest exists for either of this paper’s authors.

## Authors’ contributions

OC conceived the review paper idea. Both DW and OC participated in designing and structuring the literature search and resulting review paper structure. DW conducted the literature search, as well as the drafting of the manuscript. OC guided the literature search and drafting of the manuscript. Both authors read and approved the final manuscript prior to submission.

## Authors’ information

OC received his B.S. and M.S. degrees in Mechanical Engineering in 2004 and 2006 respectively, from Istanbul Technical University, Turkey. He completed his Ph.D. in Mechanical Engineering in 2011 at Rice University and was a Research Assistant at the Mechatronics and Haptic Interfaces (MAHI) Lab from 2006 to 2011. He was an Assistant Professor at San Francisco State University from 2011 to 2013. He is currently an Assistant Professor at Colorado School of Mines and Director of the Biomechatronics Research Laboratory. He was a recipient of the best paper award at the IEEE World Haptics Conference in 2011. His research interests include robotic rehabilitation, biomechatronics, robotics, haptics, human sensorimotor control system, motor adaptation and learning.

DW received his B.A. in Music from the University of Georgia in 2009 and his M.S. in Computer Science from San Francisco State University in 2013. Between 2012–2013, he was a Research Assistant in the School of Engineering’s Biomechatronics Research Laboratory. His research interests include computer vision, human-computer interaction, biomechatronics, robotic rehabilitation, and kinematic modeling.
